# Increasing the Utilisation of Transcranial Magnetic Stimulation for Treatment-Resistant Depression

**DOI:** 10.1192/j.eurpsy.2025.2133

**Published:** 2025-08-26

**Authors:** Y. H. Tay

**Affiliations:** Psychological Medicine, Khoo Teck Puat Hospital, Singapore, Singapore

## Abstract

**Introduction:**

Transcranial magnetic stimulation (TMS) has been consistently recommended in international guidelines as first-line for treatment-resistant depression, due to its superior efficacy over the next antidepressant, with minimal side effects. However, owing to its high cost, lack of insurance coverage, the need for daily time commitment over a period of 4 to 6 weeks and its relative novelty, TMS is still not being offered to many eligible patients with MDD including at our hospital, leading to an under-utilisation of the service.

**Objectives:**

We aim to determine the rate at which TMS is being offered to eligible patients with MDD at our hospital in Singapore, explore the root causes behind why it is not being offered and utilised more often, and implement new models of care to increase this rate by at least two-fold over 6 months.

**Methods:**

All patients who registered at our outpatient clinics from June to November 2024 were screened for the eligibility criteria for TMS. Flow charts, affinity diagram and fishbone diagram were drawn. Multi-voting was conducted to arrive at the top root causes for the low rate at which TMS was being offered to eligible depressed patients, determined on a Pareto chart. 5 Plan, Do, Study, Act cycles were carried out. Key interventions included: (a) Adopting evidence-based, shorter TMS protocols that reduced time to remission from 6 weeks to 2-3 weeks, (b) initiating a novel, nurse-led TMS counselling service, (c) improving promotional materials, and (d) using simple yet effective strategies to improve familiarity with TMS and encourage psychiatrists to consider it more often.

**Results:**

The rate at which TMS was offered to eligible patients with MDD increased from 15% to 80% over June to November 2024. 31% of these eligible patients took up TMS, and 100% of those who started TMS eventually completed the course. Patients who completed the course had a 69% reduction in clinician-administered depression rating scales. Psychiatrists were more inclined to offer TMS via the TMS counselling service run separately by TMS nurses, as this otherwise often took up substantial time in busy clinic settings. The increased awareness of TMS through promotional materials empowered patients such that they were bringing up TMS during consultations before they were being offered. The evidence-based, shorter TMS protocols involved conducting multiple sessions a day with adequate intervals, without compromising on efficacy and safety. Patients enjoyed the option of having less interruptions from work or school, and the quicker times to remission. Direct cost savings were observed.

**Conclusions:**

We observed better quality of clinical care, increased patient, staff and stakeholder satisfaction, cost and time savings to patients, and increased productivity in both patients and staff, through effective and sustainable interventions which can be replicated in TMS clinics elsewhere.

**Disclosure of Interest:**

None Declared

